# Influence of medications on thyroid function in dogs: An update

**DOI:** 10.1111/jvim.16823

**Published:** 2023-07-27

**Authors:** Timothy A. Bolton, David L. Panciera

**Affiliations:** ^1^ Department of Small Animal Clinical Sciences Virginia‐Maryland College of Veterinary Medicine Blacksburg Virginia USA

**Keywords:** amiodarone, aspirin, clomipramine, glucocorticoids, phenobarbital, sulfonamides

## Abstract

Erroneous thyroid function test results can occur because of drugs that alter thyroid hormone physiology in one or more aspects, including synthesis, secretion, distribution, and metabolism. Research since publication of the last review in the Journal of Veterinary Internal Medicine (JVIM) 20 years ago has evaluated the effects of amiodarone, zonisamide, inhalant anesthetics, clomipramine, trilostane, and toceranib on thyroid function tests in the dog. In addition, recent work on the effects of glucocorticoids, sulfonamides, phenobarbital, and nonsteroidal anti‐inflammatory drugs will be reviewed. Awareness of these effects is necessary to avoid misdiagnosis of hypothyroidism and unnecessary treatment.

**Abbreviations**
fT3: free T3
fT4: free thyroxine
HPT: hypothalamic‐pituitary‐thyroid
NSAIDs: nonsteroidal anti‐inflammatory drugs
rT3: reverse T3
T3: 3,5,3′‐triiodothyronine
T4: thyroxine
TCAs: tricyclic antidepressants
TRH: thyrotropin‐releasing hormone
TSH: thyroid‐stimulating hormone
TT3: total T3
TT4: total thyroxine

## 1 INTRODUCTION

The diagnosis of hypothyroidism is confirmed by thyroid function tests in a dog with compatible clinical and clinicopathologic abnormalities. Serum total thyroxine (TT4) or free thyroxine (fT4) hormone concentration or both below and serum thyroid‐stimulating hormone (TSH) concentration above their respective reference intervals indicate a diagnosis of primary hypothyroidism. However, no single thyroid function test is completely reliable, and approximately 25% of dogs with primary hypothyroidism have normal serum TSH concentrations.^1^, ^2^, ^3^, ^4^ Furthermore, age,^5^, ^6^ reproductive status,^7^ breed,^8^, ^9^, ^10^ body condition,^11^, ^12^ exercise or training,^13^, ^14^ nonthyroidal illness,^1^, ^2^, ^15^, ^16^ and drugs^17^ impact thyroid function test results.

Drugs affect thyroid hormone physiology in several possible ways, including synthesis, secretion, distribution, and metabolism. The administration of many drugs results in mild changes in thyroid function, whereas hypothyroidism can be induced during treatment with others. Although the effects of some drugs on thyroid function tests in dogs are well documented, the introduction of new drugs and additional research on older drugs have resulted in new findings that have relevance to clinical practice since the last review of this topic in the JVIM.^17^ Understanding the impact that various drugs have on thyroid function tests accomplishes 2 clinically applicable objectives: (1) prevents the inappropriate diagnosis of hypothyroidism and subsequent prescription of unnecessary thyroid hormone treatment, and (2) establishes when accurate thyroid hormone testing can be conducted after drug discontinuation. This review will provide an update on the effects of drugs that alter thyroid function tests in dogs, including those found in the previous review article^17^ and those studied during the intervening 20 years.

## 2 PHYSIOLOGY OF THE HYPOTHALAMIC‐PITUITARY‐THYROID AXIS

### 2.1 Hypothalamic‐pituitary‐thyroid axis overview

**FIGURE 1:**
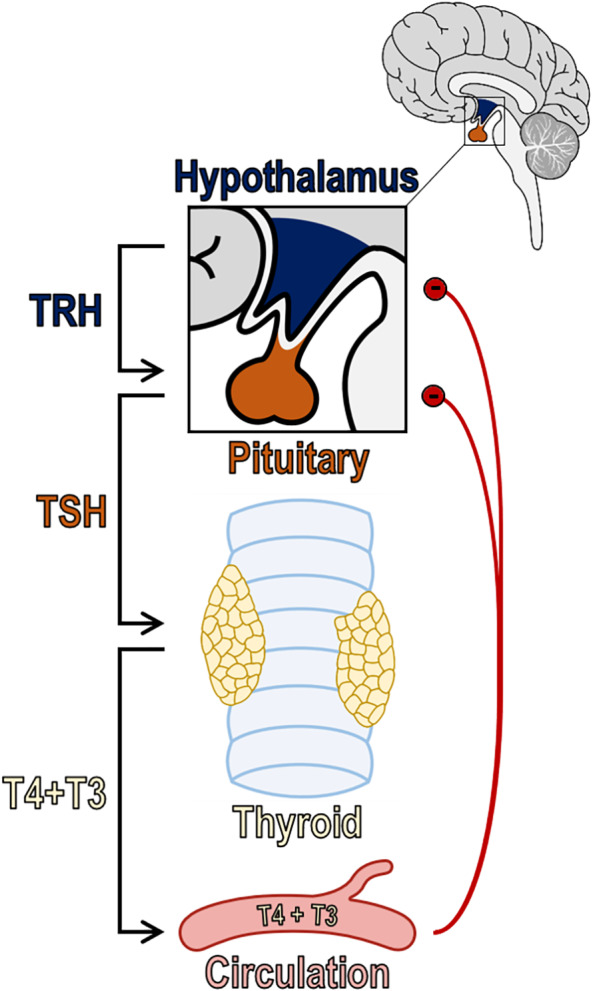
The HPT axis and its regulation. The release of TRH from the hypothalamus stimulates the secretion of TSH from the anterior pituitary gland. Following its release, TSH stimulates the synthesis and secretion of T4 and T3 from thyroid gland follicular cells into systemic circulation. The secretion of TRH and TSH is regulated by a direct negative feedback system, with circulating T4 and T3 serving as the primary inhibitors (red lines with a minus sign)

Serum thyroid hormone concentrations are determined by the interaction of the hypothalamus, anterior pituitary gland, and thyroid gland, referred to as the hypothalamic‐pituitary‐thyroid (HPT) axis (Figure 1). Thyrotropin‐releasing hormone (TRH) produced in the hypothalamus serves as the principal agonist for the secretion of TSH from the anterior pituitary gland. After release into the hypophyseal‐pituitary portal system, TRH stimulates the synthesis and secretion of TSH from thyrotropes in the anterior pituitary gland.^18^ After release into systemic circulation, TSH stimulates all steps involved in the synthesis and secretion of thyroid hormones. Additionally, TSH exerts a trophic effect, inducing the proliferation and differentiation of thyrocytes.^18^

An intricate negative feedback system, with thyroid hormones inhibiting secretion of TRH and TSH, is the primary factor controlling the HPT axis (Figure 1). It is via this pathway that plasma thyroid hormone concentrations are maintained within a narrow range. A multitude of other factors play lesser but still important roles in TRH and TSH secretion. For example, hormones related to nutrition, metabolism, and thermogenesis regulate TRH release (catecholamines and ⍺‐melanocyte‐stimulating hormone are stimulatory; leptin and neuropeptide Y are inhibitory).^18^, ^19^ The release of TSH is stimulated by catecholamines, whereas glucocorticoids, somatostatin, and dopamine inhibit its release.^18^, ^20^

### 2.2 Thyroid hormone synthesis and storage

**FIGURE 2:**
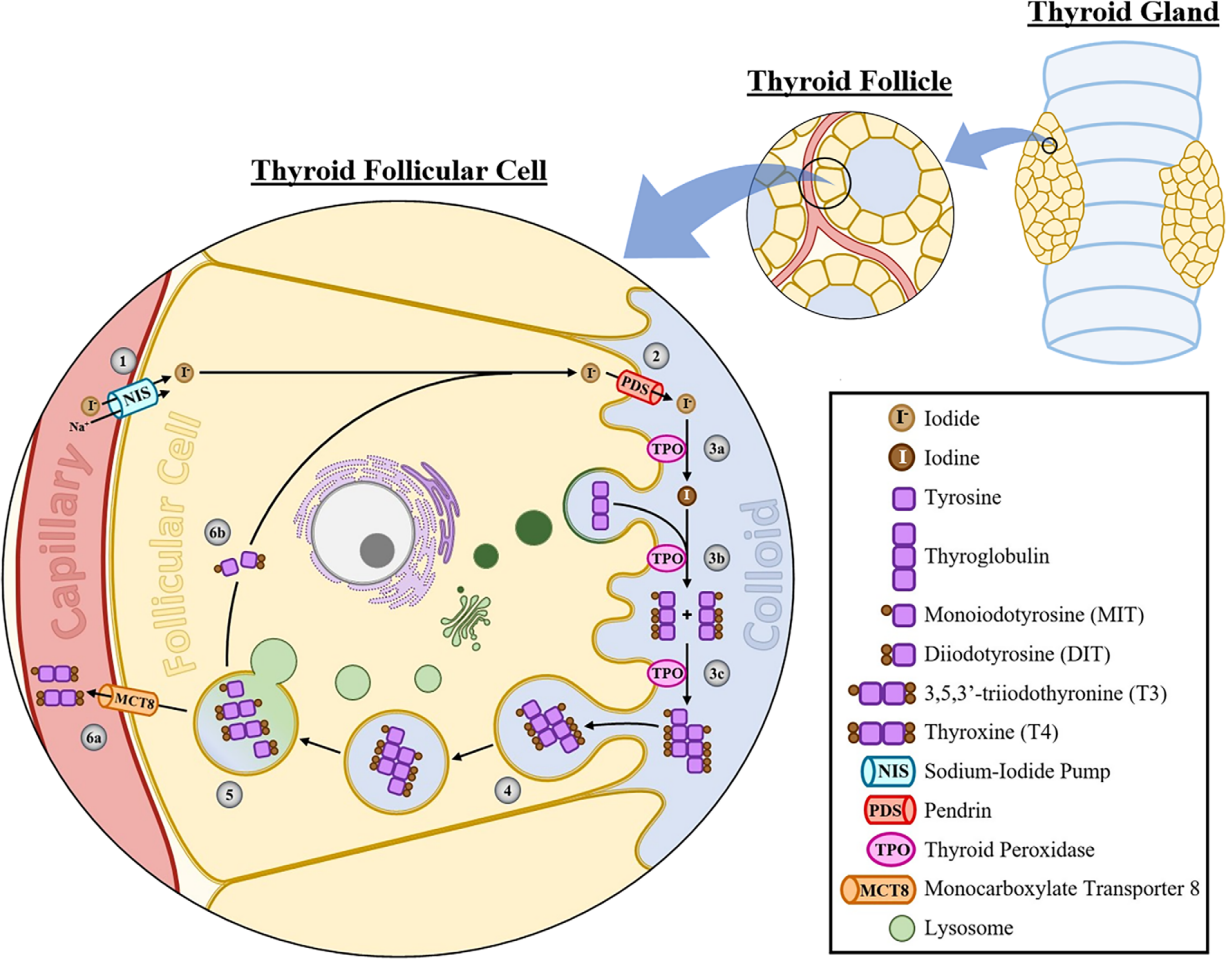
A thyroid follicular cell demonstrating the main steps of thyroid hormone synthesis and secretion. (1) In the basolateral membrane, circulating iodide is actively concentrated in the thyroid follicular cell by NIS. (2) Following diffusion across the cytosol, iodide enters the colloid via PDS. In the apical membrane, TPO catalyzes (3a) oxidation of iodide to iodine, (3b) binding of iodine to thyroglobulin (organification), generating MIT and DIT, and (3c) coupling of MIT and DIT to form T3 and T4. (4) Colloid‐containing thyroglobulin is endocytosed by the thyroid follicular cell. (5) Proteolytic enzyme‐containing lysosomes fuse with vesicles ferrying thyroglobulin, resulting in T4, T3, DIT, and MIT release. (6a) Thyroid hormones are secreted into circulation via MCT8. (6b) Iodine within DIT and MIT is recycled to be used for the organification of new thyroglobulin molecules.

Thyroid hormone synthesis occurs through a series of steps within the thyroid follicle (Figure 2). Dietary iodide absorbed from the proximal gastrointestinal tract is concentrated in the thyroid follicular cell by the sodium iodide symporter located in the basolateral membrane.^18^, ^21^, ^22^ Iodide diffuses to the follicular cell apical membrane and enters the colloid‐containing follicular lumen through the membrane‐bound protein, pendrin.^18^, ^21^ Thyroid peroxidase on the apical membrane of the follicular cell catalyzes the oxidation of iodide to iodine, binding of iodine to tyrosine residues on thyroglobulin (termed organification), and coupling of monoiodotyrosine and diiodotyrosine to produce thyroxine (T4) and 3,5,3′‐triiodothyronine (T3).^18^, ^21^, ^22^ Thyroglobulin serves as the storage depot for synthesized thyroid hormones within the follicular lumen.

The thyroid gland possesses the capacity to efficiently utilize iodine when it is both scarce and in abundance. In the latter scenario, typically a result of excess iodide ingestion, organification of iodide by thyroid peroxidase is inhibited through an intrathyroidal regulatory mechanism called the *Wolff*‐*Chaikoff* effect. In essence, iodide regulates its own organification, thereby preventing unnecessary synthesis and secretion of large quantities of thyroid hormones. In humans, this effect is sustained for several days, and then, via an escape phenomenon, organification of iodide resumes, and T4 and T3 synthesis returns to normal.^23^ Iodine‐containing drugs such as amiodarone induce hypothyroidism in humans through an inability of the thyroid gland to escape the *Wolff*‐*Chaikoff* effect.^24^

### 2.3 Thyroid hormone secretion

Similar to thyroid hormone synthesis, secretion transpires through a sequence of steps within the thyroid follicle (Figure 2). Thyroglobulin in the follicular lumen enters the follicular cell by endocytosis. In the follicular cell cytosol, vesicles transporting thyroglobulin fuse with proteolytic enzyme‐containing lysosomes, resulting in T4 and T3 release. Thyroid hormones then are secreted into systemic circulation through the follicular cell basolateral membrane. Monoiodotyrosine and diiodotyrosine also are released from thyroglobulin, but iodine contained in these molecules is removed by the enzyme, dehalogenase.^18^, ^21^ The liberated iodine is recycled within the follicular cell for organification of new thyroglobulin molecules.^18^, ^21^

### 2.4 Thyroid hormone binding and transport

Because of their hydrophobicity, thyroid hormones are reversibly bound to plasma proteins after secretion.^18^ The principal plasma protein carriers of T4 and T3 are thyroxine‐binding globulin, transthyretin, and albumin, with the remainder bound to other proteins such as immunoglobulins and lipoproteins.^18^, ^25^ Over 99% of T4 and T3 are bound to these numerous plasma proteins,^18^ whereas <1% of thyroid hormone is unbound (free) and leaves circulation to exert effects within the tissues. The protein‐bound and free thyroid hormones are in equilibrium.

### 2.5 Thyroid hormone metabolism

Thyroxine, the main secretory product of the thyroid gland, is considered a prohormone that is activated by conversion to the metabolically more potent T3.^18^, ^26^ Triiodothyronine generation is crucial because the affinity of the nuclear thyroid hormone receptor for T3 is 10‐fold higher than it is for T4.^18^, ^26^ Iodothyronine deiodinase enzymes in peripheral tissues such as the central nervous system, liver, kidneys, muscle, and skin are responsible for conversion of T4 to T3.^18^ More specifically, T3 is generated by removal of the outer ring (5′) iodine (5′‐deiodination) and the metabolically inactive reverse T3 (rT3) is produced by removal of the inner ring (5) iodine (5‐deiodination).^18^, ^26^ Thus, T4 metabolism to T3 or rT3 via iodothyronine deiodinase enzymes dictates the biological activity of thyroid hormones. Additional metabolic pathways promoting the inactivation and elimination of thyroid hormones include hepatic glucuronidation and sulfation followed by excretion in the bile and urine.^18^

### 2.6 Thyroid hormone effects

After active transport across the cell membrane, thyroid hormones exert their effects at the gene transcription level by binding receptors in the nucleus.^18^ Because thyroid hormone receptors are expressed in virtually all tissues, the myriad effects of a single hormone on such tissues highlight its fundamental role in cellular function. To name a few, thyroid hormones are essential for normal development of the nervous and skeletal systems, metabolism of macronutrients, maintenance of cardiac output, and regulation of the synthesis and secretion of several pituitary hormones (growth hormone, TSH, and prolactin).^18^, ^22^

### 2.7 Hypothyroidism in dogs

Primary hypothyroidism in the adult dog is typically the result of either idiopathic thyroid gland atrophy or autoimmune (lymphocytic) thyroiditis.^27^ It is seldom the result of thyroid gland neoplasia. Clinical and clinicopathologic abnormalities in hypothyroid dogs are the consequence of thyroid hormone deficiency on energy and nutrient metabolism (lethargy, weight gain, hypertriglyceridemia, and hypercholesterolemia), skin (alopecia, seborrhea, and pyoderma), heart (bradycardia), and nervous system (lower motor neuron, peripheral, and central vestibular disease, laryngeal paralysis, and megaesophagus).^28^, ^29^, ^30^, ^31^ Restoration of euthyroidism by PO levothyroxine treatment results in the resolution of these clinical and biochemical alterations.

## 3 EFFECTS OF DRUGS ON THYROID FUNCTION

Because species differences in thyroid hormone physiology exist, drug–thyroid interactions differ between humans and dogs. A list of medications known to impact thyroid function in these species, classified by mechanism of action, is provided (Table 1).

**TABLE 1:** Medications that affect thyroid function tests in humans (based on Davies 1991,^32^ Curran 1991,^33^ Ananthakrishnan and Pierce 2010^34^) and dogs

Mechanism of action	Medications affecting humans	Medications affecting dogs
Decreased TSH secretion	Bexarotene, dobutamine, dopamine, glucocorticoids, metformin, octreotide, TCAs	Glucocorticoids, TCAs (clomipramine)
Increased thyroid hormone secretion	Amiodarone, radiocontrast agents	None**
Decreased thyroid hormone secretion	Aminoglutethimide, amiodarone, ethionamide, lithium, propylthiouracil, radiocontrast agents, TCAs, TK inhibitors	Amiodarone, phenobarbital, sulfonamides, TCAs (clomipramine), TK inhibitors (toceranib), zonisamide*
Decreased thyroid hormone absorption	Aluminum hydroxide, calcium carbonate, cholestyramine, colestipol, ferrous sulfate, sevelamer hydrochloride, sucralfate	None**
Altered serum transport (via increased TBG concentration)	Clofibrate, estrogens, mitotane, SERMs, 5‐fluorouracil	None**
Altered serum transport (via decreased TBG concentration)	Androgens, danazol, glucocorticoids, l‐asparaginase, niacin	None**
Altered binding to serum proteins	Furosemide, heparin, NSAIDs (phenylbutazone, salicylates)	NSAIDs (aspirin, carprofen)
Increased hepatic metabolism	Carbamazepine, phenobarbital, phenytoin, rifampin	Phenobarbital
Decreased 5′‐deiodinase activity	Amiodarone, glucocorticoids, inhalation anesthetics, propranolol, propylthiouracil, radiocontrast agents	Amiodarone, glucocorticoids

Abbreviations: NSAIDs, nonsteroidal anti‐inflammatory drugs; SERMs, selective estrogen receptor modulators; TBG, thyroxine‐binding globulin; TCAs, tricyclic antidepressants; TK, tyrosine kinase.

* Refer text for more detailed explanation of this result.

** None means that studies are very limited or lacking.

Since the last review of the effects of drugs on thyroid function tests in dogs in the Journal, the impact of nonsteroidal anti‐inflammatory drugs, inhalant anesthetics, zonisamide, clomipramine, toceranib, imepitoin, and trilostane have been studied (Table 2). Additionally, newer research on glucocorticoids and sulfonamides has added to an understanding of how these drugs influence thyroid hormones based on different routes of administration and dose (Table 2). Recognizing and understanding the effects of drugs on thyroid function tests are important to clinical practice.

**TABLE 2:** Effect of drugs on canine thyroid function test results

Drug	Dose administered	Treatment duration	TT4	TT3	fT4	TSH
Prednisone (anti‐inflammatory)^35^, ^36^, ^37^, ^38^	1.0‐1.1 mg/kg/day	3^36^, ^38^ or 5^35^, ^37^ weeks	= or ↓*	↓	=	=*
Prednisone (immunosuppressive)^36^, ^39^, ^40^, ^41^, ^42^	2.2‐4.0 mg/kg/day	1^39^ or 3^36^, ^40^, ^41^, ^42^ weeks	↓	↓	↓	=
Dexamethasone (anti‐inflammatory)^43^	0.1 mg/kg q24h	3 weeks	↓	Not studied	Not ED	=
Dexamethasone (immunosuppressive)^44^	0.5‐0.66 mg/kg/day	1 day	=	↓	Not studied	Not studied
Phenobarbital (short‐term)^40^	3.6‐9.0 mg/kg/day	3 weeks	=	Not studied	=	=
Phenobarbital (long‐term)^45^, ^46^, ^47^, ^48^, ^49^, ^50^	1.0‐16.4 mg/kg/day	18,^50^ 27–29,^45^, ^47^, ^48^ or 52‐54^46^, ^49^ weeks	↓	=	↓ or =	↑ or =
Potassium bromide^45^, ^51^	30 mg/kg q24h^51^	6^51^ or 14.5^45^ months	=	=	=	=
Zonisamide^52^	10 mg/kg q12h	8 weeks	=	Not studied	=*	=*
Imepitoin^50^	30 mg/kg q12h	18 weeks	=	=	=	=
Aspirin^53^, ^54^	50^54^ or 75^53^ mg/kg/day	1^54^ or 4^53^ weeks	↓	↓	↓ or =	=
Carprofen^55^, ^56^	3.4‐6.6 mg/kg/day	5^55^ or 8^56^ weeks	↓ or =	Not studied	=	↓ or =
Sulfonamides (low dose)^57^, ^58^	Approximately 15 mg/kg q12h	3^57^ or 4^58^ weeks	↓ or =	=	↓ or =	↑
Sulfonamides (high dose)^59^, ^60^	Approximately 30 mg/kg q12h	6 weeks	↓	=	Not studied	↑
Inhalant anesthetics^61^, ^62^, ^63^		1,^62^ 2,^63^ or 14^61^ days	↓	↓ or =	↑ or =	=
Propranolol^64^	60‐120 mg/day	4 weeks	=	=	Not studied	Not studied
Clomipramine^65^	3 mg/kg q12h	112 days	↓	↑	↓	=
Toceranib^66^, ^67^	1.5‐3.0 mg/kg q48h	12 weeks	=	=	↓*	↑
Trilostane^68^		6 months	=	Not studied	↓	↑
Amiodarone^69^, ^70^	3.9‐36.4 mg/kg/day	28^69^ or 75‐84^70^ days	↑ or =	=	Not studied	=

Abbreviations: ↓, decreased; =, unchanged; ↑, increased; TT4, total T4; TT3, total T3; fT4, free T4; TSH, thyroid‐stimulating hormone; ED, equilibrium dialysis.

* Refer text for a more detailed explanation of this result.

Serum fT4 concentration results from studies using assays other than equilibrium dialysis have been excluded from this review. Dialysis assays remove antibodies and hormone‐binding proteins, thereby providing a fT4 measurement unadulterated by these compounds. Furthermore, equilibrium dialysis is the only accurate method for reporting fT4 measurement in the dog because no other fT4 assay has been shown to be accurate in pathologic conditions.^71^

Unless otherwise specified, any serum thyroid hormone result stated to be increased or decreased is significant (p <.05). No change, unchanged, and not affected mean the result is insignificant. Readers should be aware that serum thyroid hormone results that are significantly different could remain within or extend beyond the reference interval.

### 3.1 Glucocorticoids

Studies in humans have established that exogenous and endogenous glucocorticoids impact thyroid hormone physiology through 2 principal mechanisms: (1) suppression of the HPT axis; and (2) perturbation of peripheral thyroid hormone metabolism.^32^ Thyroid‐stimulating hormone secretion decreases after glucocorticoid administration, an effect mediated principally via suppressed TRH secretion, although a direct effect on TSH secretion likewise occurs.^72^, ^73^, ^74^, ^75^, ^76^, ^77^, ^78^, ^79^ Also, glucocorticoids inhibit peripheral conversion of T4 to T3 and metabolism of rT3 by iodothyronine deiodinases.^80^ The consequence of these mechanisms is a decrease in serum TT4, fT4, total T3 (TT3), and TSH concentrations and increase in serum rT3 concentration, all of minor magnitude.^32^

Prospective controlled^35^, ^36^, ^39^, ^40^, ^41^ and uncontrolled^37^, ^38^, ^42^, ^43^, ^44^, ^81^ studies in dogs have evaluated the influence of glucocorticoids on thyroid function tests. Glucocorticoids administered included prednisolone,^36^, ^42^ prednisone,^35^, ^37^, ^38^, ^39^, ^40^, ^41^ and dexamethasone.^43^, ^44^, ^81^ Although it has been known for decades that prednisone is converted to its active metabolite prednisolone after PO administration in the dog, it was recently demonstrated to be rapid (≤30 minutes) and robust (6‐fold higher plasma prednisolone concentration compared to plasma prednisone concentration) in a small group of healthy dogs.^82^ Given these findings, it is unlikely that the type of glucocorticoid (prednisone versus prednisolone) administered in studies of dogs^35^, ^36^, ^37^, ^38^, ^39^, ^40^, ^41^, ^42^ had an impact on thyroid function test results. Consequently, in the remainder of this section, prednisone will refer to either prednisone or prednisolone.

Prednisone administered PO to euthyroid dogs at an anti‐inflammatory dose (1.0‐1.1 mg/kg/day) for 3 to 5 weeks resulted in decreased serum TT3^35^, ^36^ and unchanged serum TT4^35^, ^36^ and fT4^35^ concentrations. A similar dose and duration of prednisone administered PO to thyroxine‐supplemented thyroidectomized^35^, ^37^ and hypothyroid^38^ dogs resulted in decreased serum TT3^35^, ^37^ and unchanged serum fT4^35^, ^37^, ^38^ concentrations, but unchanged^35^, ^37^ and decreased^38^ serum TT4 concentrations were found. Variable results for the serum TT4 concentration findings could be explained by study differences, such as the route of thyroxine supplementation (SC^35^, ^37^ vs PO^38^) and functionality of the thyroid gland (euthyroid^35^ vs hypothyroid^38^). The serum TSH concentration was not affected in the solitary study evaluating the hormone.^38^

A single immunosuppressive dose (2.2 mg/kg) of prednisone administered IM to dogs caused a decrease in the serum TT3 concentration 1 day after injection, whereas the serum TT4 concentration was unaltered.^39^ An immunosuppressive dose (2.2‐4.0 mg/kg/day) of prednisone administered PO or IM for a maximum of 3 weeks produced decreases in serum TT4,^36^, ^39^, ^40^, ^41^, ^42^ TT3,^36^, ^39^, ^41^, ^42^ and fT4^40^ concentrations. After 24 hours of treatment, serum TT3, TT4, and fT4 concentrations decreased by 40%, 33%, and 24%, respectively, with the latter 2 hormones decreasing further (62% and 53%, respectively) by the third week of treatment.^42^ Despite decreases in thyroid hormones, serum TSH concentration was unchanged^40^ and the T4 and T3 response to exogenous TSH administration was decreased^42^ in the only studies evaluating each of these variables.

Dexamethasone administered IM at an anti‐inflammatory dose (0.1 mg/kg/day) to dogs resulted in a decreased serum TT4 concentration after 1 week, whereas the serum TSH concentration was not affected throughout the 3‐week trial period.^43^ A single immunosuppressive dose of dexamethasone administered IM resulted in a decrease in the serum TT3 concentration 24 hours after the injection, whereas the serum TT4 concentration remained unchanged.^44^ Duration of treatment may explain the variation in serum TT4 concentration between studies.

Although the majority of veterinary studies investigating the effects of glucocorticoids on thyroid function tests in dogs have focused on systemic administration, topical administration likewise has been evaluated. Topical administration of dexamethasone to the skin of healthy dogs decreased serum TT4 and TT3 concentrations by 50% and 15%, respectively, by the conclusion of a 3‐week treatment period.^81^ Ototopical dexamethasone also decreased the serum TT4 concentration by 50% over the same time period, but had no effect on the serum TT3 concentration.^81^

Mechanisms causing alterations in thyroid function tests in dogs treated with glucocorticoids are likely similar to those in humans and other species. The decrease in serum TT3 concentration without alteration in serum TT4 concentration after anti‐inflammatory doses of glucocorticoids is consistent with inhibition of iodothyronine deiodinases. When glucocorticoids are administered at immunosuppressive doses, serum TT4, TT3, and fT4 concentrations all decrease, and the thyroid hormone response to exogenous TSH administration is decreased, all consistent with impaired thyroid hormone secretion. Because the anticipated increase in serum TSH concentration in response to decrements in thyroid hormones does not occur, TSH secretion could be impaired. Despite these decreases in serum TT4 and TT3 concentrations at both glucocorticoid dosing levels, the lack of change in the serum TSH^38^, ^40^ concentration at both dosing levels concurrent with a return to normal in the serum TT4 and TT3 concentrations 6 hours after exogenous TRH administration suggests an indirect (by suppressed TRH secretion) inhibitory effect on TSH release.^42^

Clinically, glucocorticoids administered PO or topically can result in substantial decreases in serum TT4, fT4, or TT3 concentrations. These alterations are most consistently observed at an immunosuppressive dose administered for at least 3 weeks, with such changes occurring as quickly as 24 hours after administration. In euthyroid dogs treated with immunosuppressive doses of prednisone for 3 weeks, drug withdrawal for at least 1 week is likely to result in a return of serum TT4 and fT4 concentrations to baseline, thus allowing accurate interpretation of test results.^40^

### 3.2 Phenobarbital

Early studies in rats indicated that phenobarbital accelerated the clearance rate of T4 from plasma by increased hepatic uptake and subsequent biliary and fecal excretion.^83^, ^84^, ^85^ Additional studies established that the augmented biliary excretion of T4 in phenobarbital‐treated rats was caused by induction of the T4 glucuronidation pathway in hepatic microsomes.^86^, ^87^, ^88^ Hepatic deiodination of T4 and T3 also is enhanced by phenobarbital because deiodinase is present in hepatic microsomes.^83^, ^89^ Interestingly, more recent studies have challenged the T4 glucuronidation pathway induction hypothesis as the primary method for thyroid hormone alterations in rats. One study demonstrated that decreases in the serum TT4 concentration occur in T4 glucuronyltransferase‐deficient rats,^90^ while concurrently proving that such a change resulted from its accumulation in tissues, particularly the liver.^91^ Finally, phenobarbital has been proven to influence the HPT axis by suppressing pituitary TSH secretion.^92^ The effect of these mechanisms is decreased serum TT4,^86^, ^87^, ^88^ fT4,^88^ and TT3^86^, ^87^ concentrations coupled with an increased^86^, ^87^, ^88^ or unchanged^92^ serum TSH concentration.

Administration of phenobarbital to humans results in thyroid hormone alterations similar to, but of substantially lesser magnitude than those observed in rats, resulting in a mild decrease^93^ or no change^94^, ^95^, ^96^, ^97^ in the serum TT4 concentration and unaltered serum TSH^94^, ^96^, ^97^ concentration. The results are consistent with similar mechanisms in the 2 species, but the greater effect on rats is likely the consequence of differences in phenobarbital dosage and susceptibility of the rat to thyroid disruption.^98^

Prospective controlled^40^, ^45^, ^46^ and uncontrolled^47^, ^48^, ^49^, ^50^ studies of phenobarbital administration in dogs generally identify sizeable and clinically relevant effects on thyroid function. Short‐term (<1 month) administration of phenobarbital to healthy dogs resulted in no change in serum TT4, fT4, or TSH concentrations.^40^ Conversely, long‐term (>1 month) administration of phenobarbital to healthy dogs and those with epilepsy resulted in decreases in serum TT4^45^, ^46^, ^47^, ^48^, ^49^, ^50^ concentration, with 15% to 75%^45^, ^46^, ^48^, ^49^, ^50^ of dogs having concentrations below the reference interval. A similar decrease in the serum fT4^45^, ^47^, ^48^ concentration was found in all but a single study,^50^ with 35% to 66%^45^, ^48^ of dogs having concentrations below the reference interval. The serum TSH concentration was either unchanged^45^, ^49^, ^50^ or increased.^46^, ^47^, ^48^ An increase in the serum TSH concentration occurred after phenobarbital treatment for 29 weeks and was preceded by decreases in serum TT4 and fT4 concentrations.^48^ Serum TSH concentrations increased above the reference interval in up to only 12% of dogs, much less frequently than decreases in thyroid hormone concentrations.^46^, ^48^ When measured, serum TT3 concentration was unchanged.^45^, ^48^, ^50^

Similar to rats and humans, decreases in serum TT4 and fT4 concentrations support enhanced metabolic clearance of T4, possibly by peripheral deiodination to T3 and subsequent hepatobiliary clearance and fecal excretion. The unaltered serum TT3 concentration, which remains static because of increased T4 deiodination to T3, supports enhanced clearance as opposed to decreased synthesis and secretion of the thyroid hormones. Additionally, the increase or lack of change in serum TSH concentration noted after serum TT4 and fT4 concentrations are suppressed combined with an absence of increased serum TSH concentration after phenobarbital withdrawal are consistent with an intact HPT axis, unlike the suppressed serum TSH concentration noted in a study of rats.^92^

Some dogs treated with phenobarbital chronically have thyroid function test results indicative of primary hypothyroidism. Furthermore, the adverse effects of phenobarbital and clinical signs of hypothyroidism intersect (eg, lethargy, weight gain), particularly early in drug administration. These parallels make it difficult to assess when to perform and how to interpret thyroid function tests in dogs given phenobarbital. Because serum thyroid hormone alterations in dogs receiving phenobarbital, namely TT4 and fT4, resolved by 5 weeks after drug discontinuation, it is recommended to wait at least 6 weeks after cessation of treatment before performing thyroid function testing.^47^ If phenobarbital withdrawal is not appropriate, as is the case in many patients requiring the drug for ongoing management of seizures, a trial of levothyroxine can be pursued if the suspicion of clinical hypothyroidism is still present.

### 3.3 Bromide

Iodine and bromide are halides, and bromide has the potential to impact thyroid function tests in animals and humans. In rats, bromide administration exerts a dose‐dependent decrease in serum TT4 concentration.^99^, ^100^, ^101^, ^102^ It does so by inducing relative iodine deficiency by interference with iodide uptake by the thyroid gland and inhibition of thyroid peroxidase activity.^103^ In addition to the functional effect, bromide acts as a goitrogen in rats, thus promoting an increase in follicle number, decrease in follicle size, and decrease in the quantity of colloid per follicle.^100^, ^101^, ^102^

Unlike in rats, bromide had no effect on serum TT4, TT3, fT4, and TSH concentrations when administered to healthy humans for 3 months.^104^ Also, prospective, controlled studies of healthy dogs^51^ and dogs with epilepsy^45^ that received bromide for 6 months^51^ and a median of 14.5 months,^45^ respectively, indicated no effect on serum TT4, TT3, fT4, and TSH concentrations.^45^, ^51^ When administered at therapeutic doses to healthy dogs and dogs with epilepsy, bromide does not impact thyroid function tests.

### 3.4 Zonisamide

Zonisamide is a sulfonamide‐based anticonvulsant. In humans, no information exists regarding its effect on serum thyroid hormone concentrations.^105^ However, a prospective, uncontrolled study of healthy dogs treated with zonisamide for 8 weeks identified no change in serum TT4 concentration.^52^ A nonsignificant decrease in serum fT4 concentration with a simultaneous nonsignificant increase in serum TSH concentration might suggest suppression of thyroid hormone secretion. Failure to reach statistical significance could be a result of small sample size in the study.^52^ Because sulfonamides can decrease serum TT4 concentration by inhibition of thyroid peroxidase, additional studies of the effects of zonisamide on thyroid function tests are warranted because the reported changes are similar to those of primary hypothyroidism.^106^

### 3.5 Imepitoin

In a prospective, uncontrolled study of healthy dogs treated with imepitoin for 18 weeks, no alterations in serum TT4, TT3, fT4, and TSH concentrations occurred.^50^ Although mean serum cholesterol concentration increased during the study period, the change was minimal and did not exceed the reference interval.^50^ Therefore, long‐term imepitoin treatment does not influence thyroid function tests in dogs or appreciably impact serum cholesterol concentrations, allowing for an accurate interpretation of test results in dogs while on this drug. We are not aware of any studies assessing thyroid function during imepitoin administration to other species.

### 3.6 Nonsteroidal anti‐inflammatory drugs

Nonsteroidal anti‐inflammatory drugs (NSAIDs) alter thyroid function tests by displacing T4 and T3 from their plasma protein carriers. Because thyroid hormones are highly protein bound, displacement causes a transient increase in circulating fT4 and free T3 (fT3) concentrations, briefly suppressing TSH secretion. Secretion of T4 and T3 decreases in response to the decrease in TSH, causing decreased thyroid hormone secretion and subsequently decreased serum TT4 and TT3 concentrations.^32^, ^107^, ^108^, ^109^, ^110^, ^111^ Free thyroxine is rapidly excreted and returns to normal in conjunction with normalization of TSH.

In humans, treatment with salicylates for ≤1 week resulted in 20% to 60% and 10% to 50% decreases in serum TT4 and TT3 concentrations, respectively,^108^, ^111^, ^112^, ^113^ but contradictory data regarding the effect on serum fT4 and fT3 concentrations exists. A 50% to 75% increase,^108^ unchanged,^113^ and 15% to 50% decrease^111^ in serum fT4 and fT3 concentrations have been reported. The serum TSH concentration decreased by 30% to 50%.^111^, ^112^, ^113^ Salicylate administration for >1 week produced a 15% to 70% decrease in serum TT4 concentration^110^, ^112^ and a 30% decrease in serum TT3 concentration.^110^ Unlike short‐term administration, long‐term treatment results in a steady state or equilibrium, such that the serum TSH concentration returns to baseline after weeks of treatment.^112^ Thyroid function tests in humans are minimally altered or unchanged by other NSAIDs, including ketoprofen, ibuprofen, etodolac, indomethacin, piroxicam, oxaprozin, and naproxen.^110^, ^111^, ^114^

Prospective studies have evaluated the effect of several NSAIDs on thyroid function tests in dogs, specifically aspirin, carprofen, etodolac, deracoxib, ketoprofen, and meloxicam.^53^, ^54^, ^55^, ^56^, ^115^, ^116^ Controlled studies investigating aspirin administration identified a decrease in serum TT4 and TT3 concentrations,^53^, ^54^ with 60%^53^, ^54^ and 13%^53^ of dogs having results lower than the reference interval, respectively. Although serum TSH concentrations were unchanged and within the reference interval,^53^, ^54^ serum fT4 concentration was decreased only on higher dose and longer duration (28 days) aspirin treatment.^53^

Carprofen and etodolac influence thyroid function tests in dogs, with discordant results. In an uncontrolled study of carprofen administered at 2.2 to 3.3 mg/kg PO q12h for 5 weeks, a decrease of 18% and 25% in serum TT4 and TSH concentrations, respectively, occurred.^55^ However, a controlled study of carprofen administered at 1.7 to 2.3 mg/kg PO q12h for 60 days found no change in serum TT4 and TSH concentrations.^56^ Neither study detected a change in serum fT4 concentration.^55^, ^56^ In the first of 2 uncontrolled studies, etodolac administered at 10.0 to 13.3 mg/kg PO q24h for 2 to 3 weeks to dogs with osteoarthritis or for postoperative pain relief caused a decrease in serum TT4 concentration and an increase in serum TSH concentration.^115^ Despite 21% of serum TT4 concentrations falling below the reference interval, serum TSH concentrations were normal.^115^ When etodolac was administered at 12.1 to 15.6 mg/kg PO q24h for 28 days, no effect on serum TT4 and TSH concentrations was observed.^116^ Neither study identified a change in serum fT4 concentration.^115^, ^116^ Different drug doses, durations of treatment, and patient health (healthy^55^, ^116^ vs osteoarthritis or orthopedic disease^56^, ^115^) could account for discrepancies found between studies.

Ketoprofen and deracoxib had no effect on serum TT4, fT4, TT3, and TSH concentrations in dogs when administered for 1 week^54^ and 4 weeks,^53^ respectively, whereas meloxicam administered for 60 days to dogs with osteoarthritis had no effect on serum TT4, fT4, and TSH concentrations.^56^

From a clinical perspective, aspirin has the most substantial influence on thyroid function tests in dogs. It has the potential to suppress serum TT4 concentration below the reference range within 24 hours,^54^ an effect that persists during treatment of at least 4 weeks' duration.^53^ Serum fT4 concentration also can be suppressed below the reference interval when aspirin is administered at a high therapeutic dosage for 2 weeks or longer.^53^ Etodolac has a potentially milder effect suppressing serum TT4 concentration, but this effect has not been noted consistently.^115^, ^116^ Furthermore, serum fT4 concentration is not affected.^115^, ^116^ Finally, administration of carprofen seems unlikely to result in clinically relevant alterations in thyroid function tests. If evaluation of thyroid status is desired while a dog is receiving an NSAID known to affect thyroid function tests (eg, aspirin), drug withdrawal for at least 7 to 14 days preceding testing is recommended, although the exact timing has not yet been established.^53^, ^54^ Measurement of serum fT4 and TSH concentrations should be included with the serum TT4 concentration, because these appear to be markedly less impacted by most NSAIDs.^53^, ^54^, ^55^, ^56^, ^115^, ^116^

### 3.7 Sulfonamides

Potentiated sulfonamides suppress thyroid hormone synthesis and secretion by inhibiting thyroid peroxidase.^106^ The subsequent decrease in serum thyroid hormone concentration stimulates the compensatory release of TSH from the pituitary gland by loss of negative feedback.^106^ Hyperplasia and hypertrophy of thyroid follicular cells (microscopic goiter) can occur because of increased serum TSH concentration.^106^ If follicular cell hyperplasia and hypertrophy are severe, gross enlargement (macroscopic goiter) of the thyroid gland develops.^106^ Interspecies variation in the thyroid gland response to sulfonamides is pronounced, with a mild alteration in humans^117^ compared to marked thyroid dysfunction in the rat^118^ and dog.^57^, ^59^, ^60^, ^119^, ^120^, ^121^, ^122^, ^123^

Numerous prospective studies in dogs have evaluated the effects of potentiated sulfonamides on thyroid function tests. In general, the collective results indicate that drug dose and treatment duration have the largest impact, but alternative variables such as the sulfonamide moiety used and drug PO bioavailability also may play roles.

When potentiated sulfonamides were administered at low dosages (approximately 15 mg/kg q12h) to healthy dogs for <1 month, variable effects on thyroid function tests were observed.^57^, ^58^ In a controlled study, trimethoprim‐sulfadiazine had no effect on serum TT4, TT3, or fT4 concentrations after 4 weeks of treatment.^58^ An uncontrolled study with trimethoprim‐sulfamethoxazole however found decreases in serum TT4 (50% of dogs below the reference interval) and fT4 (66% of dogs below the reference interval) concentrations, in conjunction with an increase in serum TSH concentration (66% of dogs above the reference interval) after 3 weeks of treatment.^57^ In addition to different study designs, the sulfonamide administered and the type of healthy dog used (research^58^ vs client‐owned^57^) were dissimilar between studies. Although a direct comparison has not been made, it appears that the specific sulfonamide moiety administered is an important factor in the potency of goitrogenesis of the drug.

Two uncontrolled studies whereby potentiated sulfonamides were administered at high dosages (approximately 30 mg/kg q12h) to healthy and diseased dogs for up to 6 weeks induced a consistent decrease in serum TT4 concentration (57%‐86% of dogs below the reference interval)^59^, ^60^ and increase in serum TSH concentration (57% of dogs above the reference interval).^60^ Serum TT4 and TSH concentrations were outside the reference intervals at 1 and 2 weeks, respectively, in at least 1 dog and at 2 and 3 weeks, respectively, in >50% of the dogs.^60^ The serum fT4 concentration was not measured in these studies.

Clinically, dogs receiving potentiated sulfonamides at low dosages (approximately 15 mg/kg q12h) for at least 3 weeks and high dosages (approximately 30 mg/kg q12h) for at least 1 week are likely to have thyroid function test results indicative of primary hypothyroidism (low serum TT4 with or without low fT4 and high serum TSH). If, however, a potentiated sulfonamide is prescribed at a low dosage for ≤2 weeks, minimal effects on thyroid function tests are expected.^59^ Consequently, clinicians should be aware of these effects and avoid testing thyroid function in a dog receiving potentiated sulfonamides at any dosage so as to prevent an incorrect diagnosis and the prescription of unnecessary thyroid supplementation.

In addition to thyroid function tests consistent with hypothyroidism, clinical hypothyroidism has been reported in dogs treated with a potentiated sulfonamide for time periods ranging from 10 to 210 days^119^, ^120^, ^121^, ^122^, ^123^ at dosages ranging from 24 to 40 mg/kg q12h.^119^, ^120^, ^121^, ^122^ Macroscopic^119^, ^123^ and microscopic^121^ goiter were confirmed in 2 and 1 case, respectively. Resolution of clinical signs and abnormal thyroid function tests occurred within 4 weeks^57^, ^60^, ^119^, ^121^, ^122^ of sulfonamide discontinuation, although a more protracted recovery of 12 to 22 weeks has been reported.^59^, ^120^ If thyroid function testing is desired, it should be performed at least 4 weeks after drug cessation.

### 3.8 Inhalant anesthetics

The effects of general anesthesia and surgery on thyroid function tests have received considerable investigation in humans. These studies indicate that surgery under general anesthesia causes a decrease in the serum TT3^124^, ^125^, ^126^, ^127^, ^128^, ^129^, ^130^, ^131^, ^132^ concentration and increase in serum rT3^124^, ^125^, ^126^, ^127^, ^128^, ^129^, ^131^, ^132^ concentration, suggesting impairment in the peripheral deiodination of T4 to T3 and rT3 to 3,3′‐diiodothyronine. Alterations in other thyroid hormones are variable, with increased,^124^, ^125^, ^126^, ^133^ unchanged,^128^, ^129^ and decreased^130^, ^132^ serum TT4 concentration, increased,^128^, ^132^ unchanged,^124^, ^125^, ^129^, ^130^ and decreased^126^ serum TSH concentration, and increased^125^, ^126^, ^128^, ^133^ and unchanged^130^ serum fT4 concentration all documented. The inconsistencies in these results are likely the consequences of study differences, such as anesthetic protocol, surgical procedure, and concurrent illnesses of the enrolled patients.

Studies evaluating the effects of anesthesia and surgery on thyroid function tests in the dog have been performed.^61^, ^62^, ^63^, ^134^ The duration of anesthesia administered ranged from 40 to 100 minutes, the frequency of thyroid function testing post‐anesthesia varied from minutes to daily, and the duration of post‐anesthesia monitoring ranged from 24 hours to 14 days.^61^, ^62^, ^63^, ^134^

One prospective controlled^61^ and 2 uncontrolled^62^, ^134^ studies have evaluated the effects of inhalant anesthetics alone on thyroid function tests in dogs. Inhalant anesthetics induced a decrease in serum TT4 concentration in 2 of the studies.^61^, ^62^ In 1 study, this decrease occurred at 2 and 4 hours, with increasing TT4 concentrations starting at 8 hours and returning to baseline at 48 hours.^61^ In the second study, inhalant anesthetics induced a decrease in serum TT4 concentration at 24 hours.^62^ Serum TT3 concentration decreased at 1 hour, with increasing TT3 concentrations beginning at 12 hours but never returning to baseline.^61^ Serum fT4,^61^ rT3,^61^ and TSH^61^, ^62^ concentrations remained unchanged. Contrary to these findings, 1 study^134^ reported transient increases in serum TT4 and TT3 concentrations at 15 minutes, similar to a study of humans.^124^ The reason for the discrepancy between this study^134^ and the others^61^, ^62^ is unclear.

Prospective controlled^61^ and uncontrolled^63^ studies assessing the addition of surgery to inhalant anesthesia in dogs identified similar alterations to the serum TT4 concentration when compared to inhalant anesthesia only, with a decrease occurring during the initial 8^61^ to 12 hours^63^ followed by increasing TT4 concentrations starting at 8 hours^61^ and returning to near baseline at 48 to 72 hours.^61^ Although conflicting changes in serum TT3 concentrations are reported (no change^61^ versus a decrease at 2 and 6 hours^63^), the serum rT3 concentration increased at 8 and 24 hours.^61^ Finally, serum fT4 concentration increased at 24 hours^61^ and 14 days,^61^ while serum TSH concentration was unchanged.^61^, ^63^

Inhalant anesthetics, with or without surgery, induce a clinically relevant and peracute effect on thyroid function test results in the dog. Consequently, testing, particularly TT4, should not be conducted during the 14 days after any anesthetic or surgical procedures because results may be inaccurate and prompt an incorrect diagnosis of hypothyroidism.

### 3.9 Propranolol

Propranolol decreases serum TT3^135^, ^136^ concentration and increases serum rT3^135^, ^137^, ^138^ concentration in a dose‐dependent manner in humans. These changes are the result of decreased production of T3 and metabolic clearance rate of rT3, both via 5′‐deiodinase inhibition.^135^, ^136^, ^138^ Serum TT4 and TSH concentrations appear to be minimally affected.^135^, ^136^, ^137^

In a prospective, controlled study, administration of propranolol to euthyroid dogs for 4 weeks did not affect serum TT4, TT3, or rT3 concentrations or the response of these hormones to TSH administration.^64^ Because the drug doses administered were higher than the typical therapeutic dosage, it appears that propranolol does not affect thyroid function tests in the dog.^64^

### 3.10 Tricyclic antidepressants (Clomipramine)

In humans and rats, tricyclic antidepressants (TCAs) alter thyroid function by inhibiting thyroid hormone synthesis, enhancing 5′‐deiodinase activity, and interfering with the HPT axis (through serotonergic and noradrenergic systems that interact with the hypothalamus, directly altering TRH release and indirectly TSH secretion).^139^ Impairment of thyroid hormone synthesis occurs through complexing of drug with iodine in the thyroid gland, therefore rendering it unavailable to thyroid peroxidase, and by direct inhibition of thyroid peroxidase.^139^ Serum TT4, TT3, and fT4 concentrations thus are suppressed, possibly culminating in iatrogenic hypothyroidism.^139^, ^140^ The induction of hypothyroidism is of particular concern in humans because it is associated with depression. The therapeutic benefits of TCAs on psychological disorders are augmented by thyroid hormone supplementation.^139^, ^141^

A prospective, uncontrolled study evaluating the effects of TCAs on thyroid function tests in the dog investigated clomipramine and documented decreases in serum concentrations of TT4, fT4, and rT3, with a persistent decrease in each hormone after 1 month of treatment.^65^ Despite these decreases, mean concentrations at each time point for each hormone remained within the reference interval. Although no evidence of hypothyroidism was detected during this study, the 35% and 38% decrease in serum TT4 and fT4 concentrations, respectively, could culminate in an incorrect diagnosis of hypothyroidism.^65^ Cautious interpretation of thyroid function tests in dogs receiving TCAs is warranted. However, because the duration of these effects on thyroid function is unknown, a clinical recommendation as to when accurate thyroid function testing can be performed after TCA discontinuation cannot be made.

A concomitant increase in serum TSH concentration would be expected in response to the decrease in serum TT4 and fT4 concentrations, but no change was found in the serum TSH concentration, both basal and 30 minutes post‐TRH administration.^65^ This finding may provide evidence for clomipramine‐induced suppression of TSH secretion, as occurs in rats and humans.^139^, ^142^

### 3.11 Tyrosine kinase inhibitors (Toceranib)

Tyrosine kinase inhibitors are a well‐documented cause of thyroid dysfunction or primary hypothyroidism in humans. Numerous mechanisms have been identified, such as impaired iodine uptake,^143^ thyroid peroxidase inhibition,^144^ thyroid gland capillary regression,^145^ induction of type 3 deiodinase activity,^145^ and destructive thyroiditis. Thus, it is recommended that thyroid function tests be performed regularly in humans receiving any tyrosine kinase inhibitor.^146^

Two recent prospective, uncontrolled studies have evaluated the effects of toceranib on thyroid function in tumor‐bearing dogs. Administration of toceranib for 12 weeks resulted in an increase in serum TSH^66^, ^67^ concentration whereas serum TT4^66^, ^67^ and TT3^67^ concentrations remained unchanged. One study found a decrease in serum fT4 concentration, but methodologic shortcomings (utilization of different fT4 assays and assigning the same reference interval to each assay despite different mean serum fT4 concentrations) confounded interpretation of the results.^67^ Although no dog in either study developed clinical signs of hypothyroidism, 19%^67^ and 0%^66^ of dogs had thyroid function test results consistent with primary hypothyroidism (low serum TT4 with or without low fT4 and high serum TSH) on at least 1 evaluation during the study. Because the previous study^67^ was underpowered to detect significance, no association between hypothyroidism and toceranib administration was identified. In people, because the mean time to development of hypothyroidism when receiving tyrosine kinase inhibitors can be as long as 50 weeks, a study investigating the effects of longer duration toceranib administration on thyroid function tests is needed to ascertain the true incidence of hypothyroidism in dogs receiving this drug.^146^, ^147^

### 3.12 Trilostane

A prospective, uncontrolled study of dogs with hyperadrenocorticism before treatment and after adequate control following 6 months of trilostane treatment indicated a decrease in serum fT4 concentration and increase in serum TSH concentration.^68^ The serum TT4 concentration was unaltered.^68^ Despite these changes, >50% of dogs had serum fT4 and TSH concentrations within the reference interval.^68^ The unexpected finding of a mean decrease of 50% in serum fT4 concentration after adequate hyperadrenocorticism control could explain the increase in serum TSH concentration by loss of negative feedback. Any direct effects of trilostane itself on the thyroid hormone changes in the study cannot be differentiated from the decrease in cortisol secretion induced by trilostane. We are not aware of any studies assessing thyroid function during trilostane administration in other species.

### 3.13 Amiodarone

Amiodarone is an iodine‐rich, class III antiarrhythmic that structurally resembles T4 and contains 37% iodine by weight.^24^ The alterations in thyroid function test results seen with amiodarone administration in humans are caused by inhibition of iodothyronine deiodinase in peripheral tissues, particularly the liver, but inhibition of T4 and T3 entry into tissues also plays an important role.^24^ The end result is an increase in serum TT4 and rT3 concentrations, and a decrease in serum TT3 concentration.^24^ Although the majority of humans remain euthyroid, 14% to 18% of patients develop thyroid dysfunction (hypothyroidism or thyrotoxicosis).^24^ The thyrotoxicosis is thought to be caused by (1) sizeable quantities of iodine released from the drug, resulting in excessive thyroid hormone synthesis and secretion or (2) direct amiodarone‐induced thyroid gland destruction, resulting in release of preformed thyroid hormones from damaged thyroid follicular cells, or both factors.^24^ Hypothyroidism occurs because of thyroid gland failure to escape the suppressive effects of excess iodine on thyroid hormone synthesis and secretion (*Wolff*‐*Chaikoff* effect).^24^ An inability to escape from the *Wolff*‐*Chaikoff* effect may be exacerbated by preexisting thyroiditis.^24^

The effect of amiodarone on thyroid function test results has been evaluated in normal and diseased dogs. In a prospective, controlled study of healthy dogs treated with amiodarone for 4 weeks, an increase in serum TT4 concentration with unchanged serum TT3 concentration was established, consistent with decreased iodothyronine deiodinase activity.^69^ A second *ex vivo* controlled study evaluating the effects of amiodarone on thyroid function test results identified inhibition of TSH‐induced secretion of T4 (73% of normal) and T3 (68% of normal).^148^ Finally, a retrospective study of dogs receiving amiodarone for treatment of arrhythmias found no change in serum TT4 and TSH concentrations after a median of 84 and 75 days of treatment, respectively.^70^ Health status, study power and design, and amiodarone dose could account for the inconsistent serum TT4 results found between studies.

Despite no reported cases of amiodarone‐induced thyrotoxicosis or hypothyroidism in the dog, periodic monitoring (appropriate frequency yet to be determined) of thyroid function tests in dogs receiving amiodarone is prudent because either abnormality can adversely influence cardiac function. Additionally, the serum TT4 concentration can be altered with amiodarone, warranting cautious interpretation of this result. Consequently, thyroid function testing preceding commencement of amiodarone treatment is recommended. If the dog is already receiving amiodarone, it should be discontinued, if possible, before thyroid function testing. Unfortunately, because the duration of amiodarone effects on thyroid function is unknown, a clinical recommendation as to the timing of testing after amiodarone withdrawal is not possible.

## 4 CONCLUSION

The list of drugs studied and found to alter thyroid function test results in dogs has increased over the last 20 years. In general, dogs receiving glucocorticoids, phenobarbital, NSAIDs (eg, aspirin), sulfonamides, inhalant anesthetics, clomipramine, toceranib, amiodarone, and trilostane should have thyroid function test results interpreted with caution. For some drugs, such as glucocorticoids and sulfonamides, drug dosage and treatment duration dictate whether a clinically relevant impact on thyroid function will occur. If feasible, dogs given medications known to affect thyroid function should not even have thyroid testing performed because it is likely to result in inaccurate test interpretation and potentially unnecessary treatment. If thyroid function testing is performed, interpretation of abnormal test results in the context of history and physical examination findings is crucial. Knowledge of the drugs that affect thyroid function test results in dogs and the duration of drug withdrawal, if known, needed for resolution of such effects are required for accurate interpretation of thyroid test results.

## CONFLICT OF INTEREST DECLARATION

Authors declare no conflict of interest.

## OFF‐LABEL ANTIMICROBIAL DECLARATION

Authors declare no off‐label antimicrobial use.

## INSTITUTIONAL ANIMAL CARE AND USE COMMITTEE (IACUC) OR OTHER APPROVAL DECLARATION

Authors declare no IACUC or other approval was needed.

## HUMAN ETHICS APPROVAL DECLARATION

Authors declare human ethics approval was not needed for this study.
